# Sensitivity and Specificity of a Prototype Rapid Diagnostic Test for the Detection of *Trypanosoma brucei gambiense* Infection: A Multi-centric Prospective Study

**DOI:** 10.1371/journal.pntd.0004608

**Published:** 2016-04-08

**Authors:** Sylvie Bisser, Crispin Lumbala, Etienne Nguertoum, Victor Kande, Laurence Flevaud, Gedeao Vatunga, Marleen Boelaert, Philippe Büscher, Theophile Josenando, Paul R. Bessell, Sylvain Biéler, Joseph M. Ndung’u

**Affiliations:** 1 INSERM UMR1094, Institute of Neuroepidemiology and Tropical Neurology, Limoges, France; 2 Programme National de Lutte contre la Trypanosomiase Humaine Africaine (PNLTHA), Kinshasa, Democratic Republic of the Congo; 3 Institut Centrafricain de la Recherche Agronomique (ICRA), Bangui, Central African Republic; 4 Médecins Sans Frontières (MSF) Operational Centre Barcelona-Athens (OCBA), Barcelona, Spain; 5 Instituto de Combate e Controlo das Tripanossomiases, Luanda, Angola; 6 Institute of Tropical Medicine Antwerp, Antwerpen, Belgium; 7 Epi Interventions Ltd., Edinburgh, United Kingdom; 8 Foundation for Innovative New Diagnostics, Campus Biotech, Geneva, Switzerland; Liverpool School of Tropical Medicine, UNITED KINGDOM

## Abstract

**Background:**

A major challenge in the control of human African trypanosomiasis (HAT) is lack of reliable diagnostic tests that are rapid and easy to use in remote areas where the disease occurs. In *Trypanosoma brucei gambiense* HAT, the Card Agglutination Test for Trypanosomiasis (CATT) has been the reference screening test since 1978, usually on whole blood, but also in a 1/8 dilution (CATT 1/8) to enhance specificity. However, the CATT is not available in a single format, requires a cold chain for storage, and uses equipment that requires electricity. A solution to these challenges has been provided by rapid diagnostic tests (RDT), which have recently become available. A prototype immunochromatographic test, the SD BIOLINE HAT, based on two native trypanosomal antigens (VSG LiTat 1.3 and VSG LiTat 1.5) has been developed. We carried out a non-inferiority study comparing this prototype to the CATT 1/8 in field settings.

**Methodology/Principal Findings:**

The prototype SD BIOLINE HAT, the CATT Whole Blood and CATT 1/8 were systematically applied on fresh blood samples obtained from 14,818 subjects, who were prospectively enrolled through active and passive screening in clinical studies in three endemic countries of central Africa: Angola, the Democratic Republic of the Congo and the Central African Republic. One hundred and forty nine HAT cases were confirmed by parasitology. The sensitivity and specificity of the prototype SD BIOLINE HAT was 89.26% (95% confidence interval (CI) = 83.27–93.28) and 94.58% (95% CI = 94.20–94.94) respectively. The sensitivity and specificity of the CATT on whole blood were 93.96% (95% CI = 88.92–96.79) and 95.91% (95% CI = 95.58–96.22), and of the CATT 1/8 were 89.26% (95% CI = 83.27–93.28) and 98.88% (95% CI = 98.70–99.04) respectively.

**Conclusion/Significance:**

After further optimization, the prototype SD BIOLINE HAT could become an alternative to current screening methods in primary healthcare settings in remote, resource-limited regions where HAT typically occurs.

## Author Summary

Early diagnosis and treatment of human African trypanosomiasis is essential for safe and effective treatment. The tests used to screen suspected patients and populations at risk are difficult to implement in remote rural settings where the disease occurs. Availability of simple, easy to use, instrument-free rapid diagnostic tests would improve screening and coverage of the population at risk and contribute to elimination of the disease. It would enable technicians with limited training and clinicians in emergency or medical wards to make rapid differential diagnosis for neurological syndromes or malaria-like illnesses. Introduction of such tests in all healthcare facilities in endemic regions would enable early detection of cases, hence reducing the time lost by patients before they get adequate and safe treatment. Treatment delay occurs when such patients attend non-specialized health centres that are unable to perform diagnosis of the disease. We evaluated a prototype rapid diagnostic test for HAT, the SD BIOLINE HAT in Angola, the Democratic Republic of the Congo and the Central African Republic. We show here that the test is as sensitive as the CATT in a 1/8 dilution and less sensitive than CATT on whole blood, although this latter difference was not statistically significant. The prototype RDT is a promising alternative for serodiagnosis of HAT.

## Introduction

Human African trypanosomiasis (HAT) or sleeping sickness is a neglected tropical disease that is endemic in remote, resource-limited regions of sub-Saharan African countries [1]. The disease is transmitted by infected *Glossina* species (tsetse fly) and presents in two different forms caused by two different trypanosome subspecies, *Trypanosoma brucei (T*. *b*.*) gambiense* and *T*. *b*. *rhodesiense* respectively. The two disease forms differ in their geographic location, transmission pattern, clinical manifestation and response to treatment [2, 3]. More than 95% of reported cases of HAT are caused by *T*. *b*. *gambiense* which is the focus of this study. Both forms of HAT evolve in two disease phases: an early or haemo-lymphatic phase or stage one, and a late meningo-encephalitic phase or stage two. During stage one, clinical signs are non-specific and can easily be confounded with a malaria-like illness, whereas neurological signs (sleep disorders, abnormal movements, gait disturbances and/or psychiatric disturbances) insidiously develop and characterize the stage two disease [4]. Control of *T*. *b*. *gambiense* HAT is dependent on diagnosis and treatment of infected individuals. Due to the chronic nature of the disease and a lack of specific symptoms during stage one, identification of cases relies on passive and active screening of patients and populations in endemic areas. Suspects identified using a screening test have to be confirmed as cases by demonstration of parasites in either blood, lymph node aspirates or the cerebrospinal fluid (CSF) by microscopy [4].

Thanks to international and national efforts, the number of cases of HAT reported to the World Health Organization (WHO) have been declining, from 37,991 in 1998 to 3,796 in 2014 [5,6,7]. Although the drop in cases is encouraging, there is concern that interest and funding of activities related to surveillance and control of the disease could go down, increasing the risk of resurgence [8]. In this context, and considering WHO’s goal of eliminating HAT by 2020 that was endorsed by the London Declaration of 2012 [9], it is of particular importance to implement novel control strategies. Elimination of the disease and sustaining elimination will require improved surveillance and coverage of the population at risk, using tools that have a higher accuracy, and easier to deploy in the settings where the disease occurs. This can be hampered by challenges in diagnosis of the disease, as current screening algorithms are neither sensitive enough, nor easy to use in peripheral health centres where patients first seek care, or in general hospital wards. Indeed, infected people can spend a long time seeking appropriate care without a correct diagnosis being made, ending up in stage two [10]. Management of the disease when it has advanced into the second stage is complicated, expensive (due to the requirement for in-patient treatment), and associated with risks of sequelae and relapses. There is therefore a great need for accurate, easy to use and affordable diagnostic tests for HAT that can be used to screen individuals presenting with symptoms with a differential diagnosis that includes HAT [11]. Such tests would enable integration of HAT diagnosis in the general primary health care system, thus improving coverage of the population at risk.

Alere/Standard Diagnostics, Inc., South Korea, in collaboration with among others the Foundation for Innovative New Diagnostics (FIND), Switzerland and the Institute of Tropical Medicine (ITM) in Belgium, has developed a number of prototype rapid diagnostic tests (RDTs) for HAT using different trypanosome antigen combinations. Comparison of the prototype RDTs with the reference antibody detection screening test in current use, the card agglutination test for trypanosomiasis (CATT/*T*.*b*.*gambiense*) [12] using stored plasma samples showed that the SD BIOLINE HAT performed as well as CATT (S1 File). A limitation of that comparison however, was that the plasma samples had been pre-selected using CATT, as it was the only screening test for HAT that was in use at the time. The aim of this study was to evaluate the diagnostic accuracy of the prototype SD BIOLINE HAT using fresh blood samples under field conditions in a cross-sectional study; to compare its performance with that of CATT, on subjects who are not pre-selected using another serological method. CATT on 1/8 diluted plasma, which is more specific than CATT on whole blood and may be more suited for the target diagnostic settings, was considered for the main comparison. The study was carried out in three countries, in order to assess whether regional differences associated with parasite strains, level of endemicity or population genetic polymorphisms would interfere with test results. Angola, the Democratic Republic of the Congo (DRC) and the Central African Republic (CAR) were selected as countries with low (0.1–0.5%), intermediate (0.5–1.5%) and high (1.5–3.0%) prevalence, respectively. Subjects were enrolled through passive screening in fixed healthcare facilities and through active screening by mobile teams.

## Methods

### Participants

The settings, prevalence, names and locations of the study sites are summarized in Table 1. Study participants were enrolled in HAT endemic regions during both active and passive screening activities by teams of the national sleeping sickness control programs in Angola, the DRC and CAR. Cases of HAT were defined as subjects in whom trypanosomes were demonstrated by microscopy in either blood, lymph node aspirate or CSF. Cases were classified as stage one when no trypanosomes were observed in CSF and when the CSF white cell count was lower than or equal to 5 cells/μL, while those with trypanosomes in CSF and/or a CSF cell count above 5 cells/μL were classified as stage two. Controls were subjects living in the same areas as cases, with no previous history of HAT treatment and who were either seronegative (negative with all serological tests), or who were seropositive (positive with one or more serological tests) but with no detectable parasites in body fluids.

**Table 1:** Sites in Angola, CAR and the DRC where participants were enrolled by both passive and active screening. Prevalence was calculated on the basis of combined active and passive screening results from this study.

Country	County/ prefecture/ province	HAT prevalence	Commune/health district	Fixed centre	Mobile team
Angola	Bengo, Uuige	0.3%	Caxito	Caxito, Uige	Mumbondo
	Kwanza Norte		Mumbondo	Ndalatando	
CAR	Batangafo	2.13%	Hama, Bakassi		Batangafo
			Ouassi		
DRC	East Kasaï	1.2%	Bibanga, Tshilenge	Tshibila	Tshilenge

CAR = Central African Republic; DRC = Democratic Republic of the Congo.

### Test procedures

**Fig 1:**
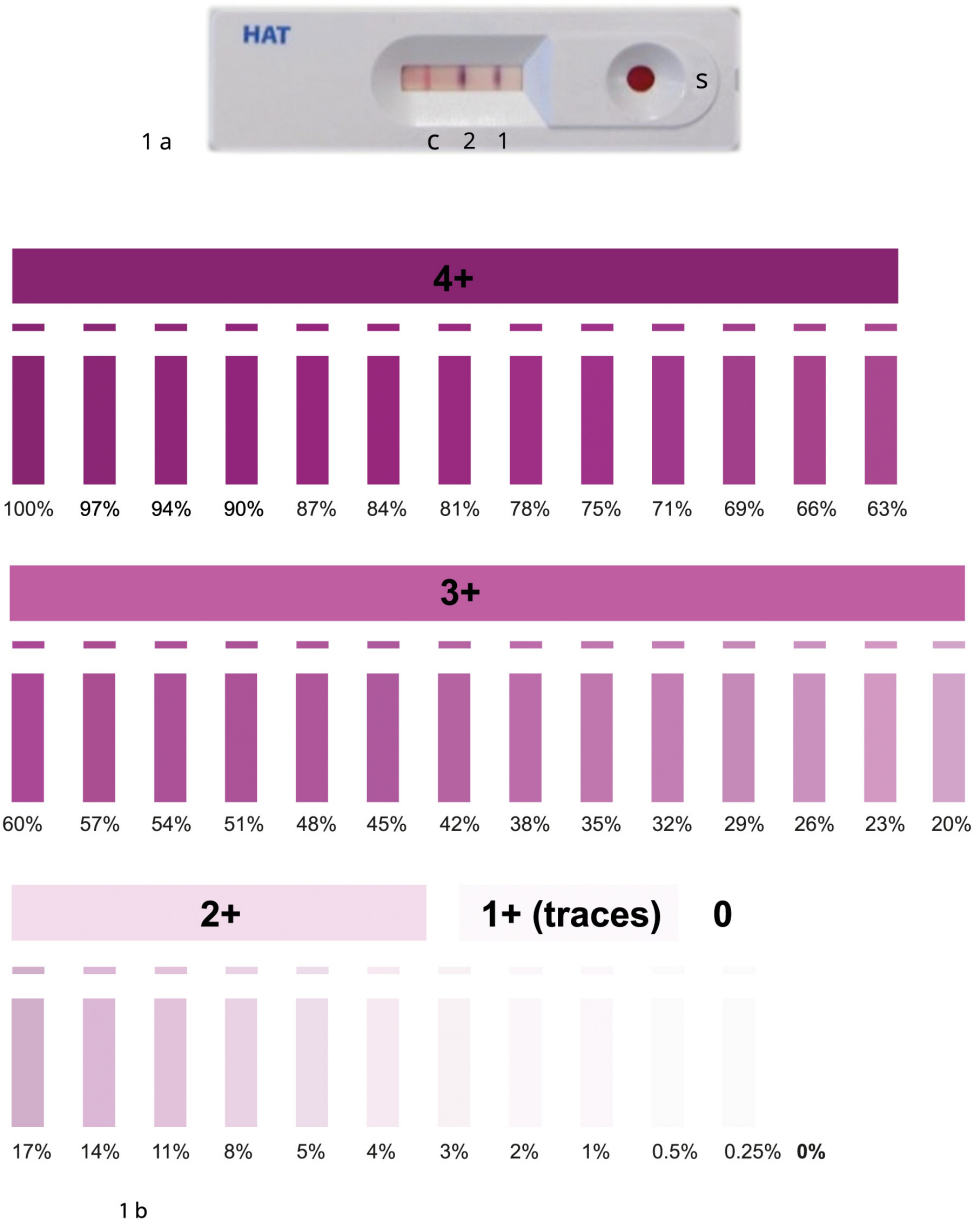
Example of an SD BIOLINE HAT positive test and the colour chart provided for interpretation of test intensity (the color chart is for research purposes only, the final test would be as positive or negative result only). 1a: from right to left: S: sample well, 1: band 1(LiTat 1.3), band 2 (LiTat 1.5). 1b: from top to bottom: possible results 4+, 3+, 2+, 1+, 0, and the range of color intensity corresponding to each result.

The prototype SD BIOLINE HAT is an immunochromatographic test for qualitative detection of antibodies of all isotypes (IgG, IgA and IgM). The test has a nitrocellulose membrane strip with two regions (T1 and T2) that are pre-coated with two native variant surface glycoprotein (VSG) antigens from *T*.*b*. *gambiense* (VSG LiTat1.3 and VSG LiTat 1.5 respectively). It also has a procedural control line (C) (Fig 1). The test is stable for at least 24 months at 40°C, or at least 5 weeks at 55°C. To perform the test, 20 μl of whole blood is taken from a finger prick and transferred into a sample well using a plastic pipette, and 4 drops (approximately 120 μl) of chase buffer are added. The sample flows along the membrane by capillarity, passing through the two test regions. Results are read after 15 to 20 minutes by comparing the intensity of the test lines against a colour chart provided by the manufacturer (Fig 1a and 1b). A result is considered positive when the control line C and either one or both test lines T1 and T2 are visible, negative when only the C line is observed and invalid if the C line is not observed. All participants found positive with the RDT or CATT test were tested for malaria using an SD BIOLINE malaria Ag P.f RDT. Those who were positive for malaria were examined, and if necessary, treated in line with national guidelines.

Both CATT and SD BIOLINE HAT were performed on finger-prick blood from each subject who presented to mobile teams and any subject who presented to a health centre with symptoms indicative of HAT. Any subject who was positive by the RDT and/or CATT or who showed symptoms highly suggestive of HAT (i.e. a combination of at least 2 neurological signs) was eligible for enrolment in the parasitological work-up. Written informed consent was sought from these subjects prior to enrolment. Any individual who declined to participate was followed up according to the standard procedures of the national control programme. Individuals who were negative in both CATT and RDT were considered as not infected and were not investigated further. A lymph node aspirate was collected from any subject who presented with swollen lymph nodes and examined for trypanosomes by microscopy. Ten ml venous blood with heparin as anticoagulant, were collected from each RDT and/or CATT positive subject, as well as those with palpable lymph nodes. Six hundred μl of blood was used to perform the capillary tube centrifugation (CTC) test (4 capillary tubes of 75 μl) and the miniature anion exchange centrifugation technique (mAECT) (300 μl) [13], except in CAR where mAECT was not performed as it was not in routine use. For subjects who were positive by CATT on whole blood, 1 ml plasma was used to perform CATT dilutions [14]. Parasitologically confirmed cases and/or subjects found positive by CATT at a dilution of 1/16 who were negative by all other parasitological methods that were performed underwent a lumbar puncture in accordance with national guidelines for stage determination and/or parasitological confirmation in CSF when there were suggestive neurological signs. Parasitological examination of CSF was done using the modified single centrifugation technique [13]. The technicians who performed the tests were part of the teams of the national sleeping sickness control programmes, with experience in performing routine parasitological tests for detection of trypanosomes. They were trained on how to perform, read and interpret results of the RDTs.

There were two levels of blinding. During the initial screening of participants using blood from a finger prick, one health worker was responsible for performing the CATT test, while another health worker tested them with the RDT. The two health workers operated independently (but used blood from the same finger prick), without exchanging results (first level of blinding). A supervisor was responsible for collecting results of the two tests and deciding whether or not to collect venous blood for the parasitological tests. Samples of venous blood were labelled with blinding codes by the supervisor (second level of blinding). The same codes were used to identify all other samples collected from the participants (e.g. the buffy coat, plasma, CSF and/or blood on filter paper) and constituted the anonymisation process kept all along the study.

All plasma, buffy coat and CSF samples that remained after the diagnostic procedures were aliquoted and stored in liquid nitrogen. Due to logistical constraints in CAR that limited access to liquid nitrogen for sample storage, blood was dried on Whatman filter paper, while plasma was stored at 4°C in the field for not more than 2 weeks before being transferred to Bangui, where it was stored at -20°C. The samples were later shipped to either Limoges University (France) or Makerere University (Uganda) for storage. Samples from a subset of subjects who were positive by any screening method but not by parasitology were later tested with two molecular methods, including the loop-mediated isothermal amplification (LAMP) developed by Eiken Chemical Co. [15] and polymerase chain reaction (PCR) using primers specific for the *T*. *brucei* group [16].

### Sample size calculation

We aimed to enrol sufficient study participants to give a robust estimate of test performance parameters whilst ensuring that the target sample size would be logistically feasible for such a prospective study. In order to achieve this, we calculated a sample size to demonstrate non-inferiority of the sensitivity and specificity of the RDT compared to the CATT 1/8 with a confidence interval (1-alpha) of 95% and a power (1-beta) of 80%, a non-inferiority margin of 5%, and using an expected sensitivity and specificity of 95% for both tests. The minimum number of study participants required to achieve this was 235 true HAT cases and 235 controls [17]. Based on the expected prevalence of HAT in the study areas, the minimum number of subjects estimated to be enrolled in order to get at least 235 HAT cases were 6,320 in Angola and 4,200 in each of the DRC and CAR, for a total of 14,720 subjects.

### Statistical analysis

Sensitivity and specificity were calculated for the prototype RDT, CATT on whole blood and CATT on 1/4 and 1/8 diluted plasma, by country, by disease stage and by screening method. In the field, CATT dilutions are sometimes performed on subjects who are negative by CATT on whole blood if the subject has symptoms indicative of HAT. However, since CATT dilutions are usually not performed on subjects who are negative by CATT on whole blood, we considered any subject who was negative by CATT on whole blood to also be negative by CATT on diluted plasma for purposes of the analysis.

The results of the serological tests CATT and RDT were each compared to a composite reference standard (CRS). This CRS classified participants based on demonstration of trypanosomes by any of the parasitological methods. A participant was classified as CRS positive, and hence considered as a HAT case, when parasites were demonstrated by any of the parasitological tests. A participant was classified as CRS negative if parasites were not identified in any body fluid, provided that at least CTC and mAECT had been performed (except in CAR where mAECT was not used).

The results on diagnostic accuracy of the tests are reported in a descriptive manner, without claiming non-inferiority for two reasons: the required sample size could not be reached as the prevalence of HAT was lower than expected and the actual sensitivity and specificity of the CATT 1/8 and the RDT were also lower than anticipated. Therefore, 95% confidence intervals (CI) were calculated around the sensitivity and specificity estimates using the Wilson method implemented in the Hmisc package for the R statistical environment [18, 19]. Statistical significance was analysed by checking for an overlap in confidence intervals and more formally using Pearson’s Chi-squared test.

In an additional analysis, we compared the results of molecular analyses to those of parasitology to identify participants who were potentially parasitological false negatives.

### Ethical approval

The study received ethical clearance from the different committees of the three participating countries respectively for DRC, CAR and Angola: “Ecole de santé publique de l’Université de Kinshasa”; “Comité scientifique de la faculté des sciences de la santé” and “direccao nacional da saude publica, Ministerio da saude”. Participants provided written informed consent before being enrolled in the study. For children below 18 years, consent was provided by a parent or guardian. All individuals who presented at study sites during the period of enrollment and consented to being screened were eligible. All participants’ samples were blinded and further analysed anonymously.

## Results

### Overview of participants and test results

**Fig 2:**
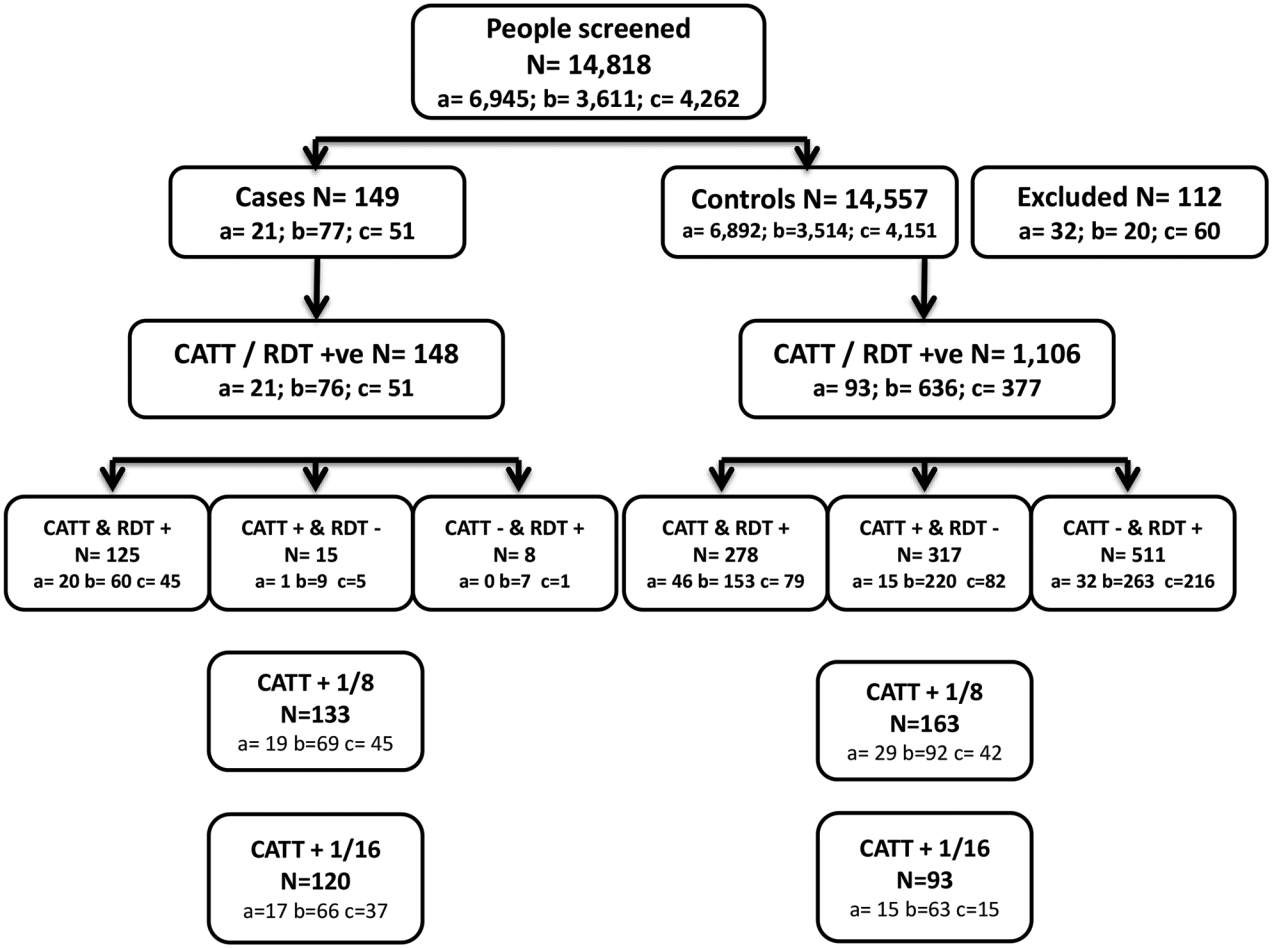
Overview of the results of the CATT and RDT tests in Angola, CAR and the DRC. RDT: SD BIOLINE HAT rapid diagnostic test. CATT: card agglutination test for trypanosomiasis. * 1 case that was negative on CATT whole blood and the RDT was detected by CATT 1/8 dilution. ** a = Angola; b = Central African Republic; c = Democratic Republic of the Congo. / = and/or

Enrollment of participants was carried out from July to October 2011 in Angola, September 2012 to March 2013 in the DRC, and from April to June 2012 in CAR. The global and country-specific overview of the results of the study is shown as a flow chart in Fig 2. In total, 14,818 participants were screened using both serological tests (9.5% in passive screening), and out of these 149 HAT cases were confirmed by parasitology. Hence, the prevalence of HAT was found to be lower than originally estimated, and as such it was not possible to reach the target number of cases. Among the remaining 14,669 participants, 112 could not be included as controls because 98 had previous history of HAT, and 14 who were positive by serology were excluded during analysis because their parasitology results were incomplete (CTC had not been performed).

Among the 149 confirmed cases, 148 were positive by either CATT on whole blood and/or RDT, while one case was negative by both CATT whole blood and RDT, and was detected by CATT dilutions. CATT dilutions were performed on this case because of the presence of symptoms suggestive of HAT. Twenty one cases were identified in Angola (detection rate of 0.30%), 77 in CAR (detection rate of 2.13%) and 51 in the DRC (detection rate of 1.20%). The median age of cases was 24 years (range 1–78) with a sex ratio of 0.84 (68 males/81 females). One hundred and nine cases (73.2%) were enrolled during active screening and 40 (26.8%) during passive screening. Forty six (31%) cases were in stage one disease and 103 (69%) were in stage two. Forty percent of cases identified by active screening were in stage one disease, compared to only 5% among cases identified by passive screening.

Among the 14,557 controls, 1,106 (7.6%) were positive with either one or both screening tests, and 13,437 were negative with both. Of these controls, 6,892 (47.4%) were enrolled in Angola (5,877 by active screening and 1,013 by passive screening), 3,514 (24.2%) in CAR (all by active screening) and 4,151 (28.5%) in the DRC (3,816 by active screening and 335 by passive screening). Among the 1,106 positive tests, 278 were positive with both serological tests, 317 were positive by CATT and negative by RDT and 511 were negative by CATT and positive by RDT. No invalid RDT result was reported from any country.

Eight hundred and twenty seven subjects (548 seropositive with at least one test and 279 positive by both tests) were tested using LAMP and PCR. Both molecular tests were negative in 664 (80.29%) samples, 65 (7.86%) were positive with LAMP only and 72 (8.71%) with PCR only. Both molecular tests were positive in 26 (3.14%) samples, 25 of which were from CAR where mAECT had not been used for parasitological confirmation of cases.

Results on sensitivity and specificity for each test and each CATT dilution by country, screening method and disease stage are shown in Tables 2, 3 and 4 respectively. The overall sensitivity of the RDT and CATT at 1/8 dilution was identical, at 89.26% (95% CI = 83.27–93.28), while that for CATT on whole blood was higher, at 93.96% (95% CI = 88.92–96.79%). Sensitivity of CATT on whole blood was highest in Angola and lowest in CAR. However, in CAR CATT retained its sensitivity when serial dilutions were considered, whilst in Angola and DRC the sensitivity declined, such that a dilution of 1/16 had the highest sensitivity in CAR (Table 2). The sensitivity of the RDT was highest in Angola and lowest in CAR. It is noticeable that the sensitivity of band 1 on the RDT, which has the same antigen as the one in the CATT test, was very similar in the three countries (85.71; 85.71 and 86.27% respectively for Angola, CAR and DRC), whilst the sensitivity of band 2 varied from 95.24% in Angola (95% CI = 77.33–99.76) to 83.12% (95% CI = 73.23–89.86) in CAR.

**Table 2:** Performance of CATT and prototype SD BIOLINE HAT RDT on fresh blood by country.

	Nb of HAT cases testing +ve	Sensitivity (95% CI)	Nb of controls testing -ve	Specificity (95% CI)
CATT WB				
**All**	**140 \ 149**	**93.96 (88.92–96.79)**	13,962 \ 14,557*	**95.91 (95.58–96.22)**
Angola	21 \ 21	100.0 (84.54–100.0)	6,831 \ 6,892	99.11 (98.86–99.31)
CAR	69 \ 77	89.61 (80.82–94.64)	3,141 \ 3,514	89.39 (88.32–90.36)
DRC	50 \ 51	98.04 (89.70–99.90)	3,990 \ 4,151	96.12 (95.49–96.67)
CATT 1/8				
**All**	**133 \ 149**	**89.26 (83.27–93.28)**	14,388 \ 14,551*	**98.88 (98.7–99.04)**
Angola	19 \ 21	90.48 (71.09–97.35)	6,863 \ 6,892	99.58 (99.4–99.71)
CAR	69 \ 77	89.61 (80.82–94.64)	3,419 \ 3,511	97.38 (96.8–97.86)
DRC	45 \ 51	88.24 (76.62–94.49)	4,106 \ 4,148	98.99 (98.63–99.25)
CATT 1/16				
**All**	**120 \ 149**	**80.54 (73.45–86.09)**	14,458 \ 14,551*	**99.36 (99.22–99.48)**
Angola	17 \ 21	80.95 (60.00–92.33)	6,877 \ 6,892	99.78 (99.64–99.87)
CAR	66 \ 77	85.71 (76.2–91.83)	3,448 \ 3,511	98.21 (97.71–98.59)
DRC	37 \ 51	72.55 (59.05–82.89)	4,133 \ 4,148	99.64 (99.40–99.78)
RDT all bands				
**All**	**133 \ 149**	**89.26 (83.27–93.28)**	**13,768 \ 14,557**	**94.58 (94.2–94.94)**
Angola	20 \ 21	95.24 (77.33–99.76)	6,814 \ 6,892	98.87 (98.59–99.09)
CAR	67 \ 77	87.01 (77.72–92.79)	3,098 \ 3,514	88.16 (87.05–89.19)
DRC	46 \ 51	90.20 (79.02–95.74)	3,856 \ 4,151	92.89 (92.07–93.64)
RDT band 1				
**All**	**128 \ 149**	**85.91 (79.41–90.59)**	**13,910 \ 14,557**	**95.56 (95.21–95.88)**
Angola	18 \ 21	85.71 (65.36–95.02)	6,829 \ 6,892	99.09 (98.83–99.28)
CAR	66 \ 77	85.71 (76.20–91.83)	3,116 \ 3,514	88.67 (87.58–89.68)
DRC	44 \ 51	86.27 (74.28–93.19)	3,965 \ 4,151	95.52 (94.85–96.11)
RDT band 2				
**All**	**128 \ 149**	**85.91 (79.41–90.59)**	**13,839 \ 14,557**	**95.07 (94.7–95.41)**
Angola	20 \ 21	95.24 (77.33–99.76)	6,818 \ 6,892	98.93 (98.65–99.14)
CAR	64 \ 77	83.12 (73.23–89.86)	3,129 \ 3,514	89.04 (87.97–90.03)
DRC	44 \ 51	86.27 (74.28–93.19)	3,892 \ 4,151	93.76 (92.98–94.46)

*Number of controls for CATT dilutions are less by 6 (14,557 versus 14,551) because these were positive by whole blood, but no dilutions were performed.

RDT: SD BIOLINE HAT rapid diagnostic test; CATT: card agglutination test for trypanosomiasis.

WB: whole blood; 1/8, 1/16 are dilutions for the CATT test; CAR: Central African Republic; DRC: Democratic Republic of the Congo.

**Table 3:** Performance of CATT and prototype SD BIOLINE HAT RDT on fresh blood by method of screening.

	Nb of HAT cases	Sensitivity	Nb of controls	Specificity
	testing +ve	(95%CI)	testing -ve	(95% CI)
CATT WB				
**All**	**140 \ 149**	**93.96 (88.92–96.79)**	**13,962 \ 14,557**	**95.91 (95.57–96.22)**
Active	100 \ 109	91.74 (85.05–95.60)	12,635 \ 13,209	95.65 (95.29–95.99)
Passive	40 \ 40	100.0 (91.24–100.0)	1,327 \ 1,348	98.44 (97.63–98.98)
CATT 1/8				
**All**	**133 \ 149**	**89.26 (83.27–93.28)**	**14,388 \ 14,551**	**98.88 (98.70–99.04)**
Active	99 \ 109	90.83 (83.93–94.94)	13,048 \ 13,203	98.83 (98.63–99.00)
Passive	34 \ 40	85.00 (70.93–92.94)	1,340 \ 1,348	99.41 (98.83–99.70)
CATT 1/16				
**All**	**120 \ 149**	**80.54 (73.45–86.09)**	**14,458 \ 14,551**	**99.36 (99.22–99.48)**
Active	88 \ 109	80.73 (72.34–87.04)	13,115 \ 13,203	99.33 (99.18–99.46)
Passive	32 \ 40	80.00 (65.24–89.50)	1,343 \ 1,348	99.63 (99.13–99.84)
RDT all bands				
**All**	**133 \ 149**	**89.26 (83.27–93.28)**	**13,768 \ 14,557**	**94.58 (94.20–94.94)**
Active	96 \ 109	88.07 (80.66–92.90)	12,450 \ 13,209	94.25 (93.84–94.64)
Passive	37 \ 40	92.50 (80.14–97.42)	1,318 \ 1,348	97.77 (96.84–98.44)
RDT band 1				
**All**	**128 \ 149**	**85.91 (79.41–90.59)**	**13,910 \ 14,557**	**95.56 (95.21–95.88)**
Active	94 \ 109	86.24 (78.53–91.48)	12,584 \ 13,209	95.27 (94.89–95.62)
Passive	34 \ 40	85.00 (70.93–92.94)	1,326 \ 1,348	98.37 (97.54–98.92)
RDT band 2				
**All**	**128 \ 149**	**85.91 (79.41–90.59)**	**13,839 \ 14,557**	**95.07 (94.70–95.41)**
Active	91 \ 109	83.49 (75.40–89.29)	12,516 \ 13,209	94.75 (94.36–95.12)
Passive	37 \ 40	92.50 (80.14–97.42)	1,323 \ 1,348	98.15 (97.28–98.74)

RDT: SD BIOLINE HAT rapid diagnostic test; CATT: card agglutination test for trypanosomiasis;

WB: whole blood; 1/4, 1/8, 1/16 are dilutions for the CATT test.

**Table 4:** Sensitivity of CATT and prototype SD BIOLINE HAT RDT on fresh blood by stage of disease.

	Nb of patients	Sensitivity
	testing +ve	(95% CI)
CATT WB		
**All**	140 \ 149	93.96 (88.92–96.79)
Stage 1	42 \ 46	91.30 (79.68–96.57)
Stage 2	98 \ 103	95.15 (89.14–97.91)
CATT 1/8		
**All**	**133 \ 149**	**89.26 (83.27–93.28)**
Stage 1	41 \ 46	89.13 (76.96–95.27)
Stage 2	92 \ 103	89.32 (81.88–93.93)
CATT 1/16		
**All**	**120 \ 149**	**80.54 (73.45–86.09)**
Stage 1	36 \ 46	78.26 (64.43–87.74)
Stage 2	84 \ 103	81.55 (72.98–87.86)
RDT all bands		
**All**	**133 \ 149**	**89.26 (83.27–93.28)**
Stage 1	38 \ 46	82.61 (69.28–90.91)
Stage 2	95 \ 103	92.23 (85.42–96.01)
RDT band 1		
**All**	**128 \ 149**	**85.91 (79.41–90.59)**
Stage 1	37 \ 46	80.43 (66.83–89.35)
Stage 2	91 \ 103	88.35 (80.73–93.21)
RDT band 2		
**All**	**128 \ 149**	**85.91 (79.41–90.59)**
Stage 1	35 \ 46	76.09 (62.06–86.09)
Stage 2	93 \ 103	90.29 (83.04–94.64)

RDT: SD BIOLINE HAT rapid diagnostic test; CATT: card agglutination test for trypanosomiasis.

WB: whole blood; 1/8, 1/16 are dilutions for the CATT test.

The overall specificity was 94.58% (95% CI = 94.20–94.94%) for the RDT, 95.91% (95% CI = 95.58–96.22%) for CATT on whole blood, and 98.88% (95% CI = 98.70–99.04%) for CATT at 1/8 dilution. There were significant differences (p<0.001) between the three countries in the specificity of all tests except for CATT 1/16 (99.36% overall specificity (95% CI = 99.22–99.48%) (Table 2). As was seen with sensitivity, all tests were most specific in Angola and least specific in CAR.

Both the RDT and CATT on whole blood were more sensitive in stage two than in stage one patients (Table 4). Similarly, both the RDT and CATT on whole blood were more sensitive in passive than in active screening (Table 3). However, when CATT at 1/8 dilution was considered, sensitivity was higher in active than in passive screening (Table 3). The specificity of all tests was higher in passive than active screening (Table 3).

If a hypothetical cohort of 1,000 subjects with a HAT prevalence of 1% is considered, then the RDT would result in 53 false positives proceeding for confirmatory testing, while CATT WB and CATT 1/8 would results in 40 and 11 false positives respectively (Table 5).

**Table 5:** Test outcomes based on a hypothetical cohort of 1,000 subjects with a HAT prevalence of 1%.

	Infected	Non-infected
CATT WB		
Pos	9.4	40.47
Neg	0.6	949.53
CATT 1/8		
Pos	8.93	11.49
Neg	1.07	978.51
RDT		
Pos	8.93	53.66
Neg	1.07	936.34

RDT: SD BIOLINE HAT rapid diagnostic test; CATT: card agglutination test for trypanosomiasis;

Neg: negative test, Pos: positive test.

Agreement between CATT on whole blood and RDT was 93.91%, with both positive for 426 (2.87%) participants (Table 6). Both tests were negative in 13,489 (91%), 361 (2.4%) were CATT positive and RDT negative, while 542 (3.6%) were CATT negative and RDT positive. Agreement increased slightly when band 1 of the RDT was considered on its own (94.53%) but was similar for RDT band 2 (93.99%).

**Table 6:** Agreement between CATT on whole blood and SD BIOLINE HAT RDT, RDT band 1 and RDT band 2. This table includes all enrolled participants.

	CATT		% Agreement
	Neg	Pos	
RDT			
Neg	13,489	361	93.91
Pos	542	426	
RDT band 1			
Neg	13,615	396	94.53
Pos	415	392	
RDT band 2			
Neg	13,535	396	93.99
Pos	495	392	

RDT: SD BIOLINE HAT rapid diagnostic test; CATT: card agglutination test for trypanosomiasis;

Neg: negative test, Pos: positive test.

## Discussion

This study is the first report of a multi-centric evaluation of an RDT for screening HAT in settings where the disease occurs in central African countries. It is the first study conducted without pre-selection of participants on the basis of their CATT status, the only screening test that was in routine used at that time. The overall sensitivity of the SD BIOLINE HAT RDT and CATT at 1/8 dilution were identical (89.26%, 95% CI 83.27, 93.28%). The RDT was less sensitive than CATT on whole blood, although this difference was not statistically significant. However, the specificity of the RDT was 4.3% lower than that of CATT at 1/8 dilution, at 94.58% (95% CI 94.20, 94.94%). Comparison of the prototype RDT to CATT at 1/8 dilution was in line with the knowledge that the specificity of CATT is improved when this dilution is considered.

The sensitivity of the prototype RDT (89.26%) was lower than that reported in a recent study in the DRC on another RDT (98.5% for the HAT Sero-*K*-SeT) [20]. This may be explained by methodological differences, as our study included both active and passive screening, none of the study participants had been pre-selected with CATT, and the level of blinding was higher. During passive screening, a patient is subjected to a screening test after being suspected of having HAT, while in active screening, all people who present themselves are screened. As a result of the clinical suspicion before being tested, a passive screening setting may therefore bias the results towards higher sensitivity. The two studies are however in agreement that a combination of at least two antigens (VSG LiTat 1.3 and VSG LiTat 1.5) on the same test is necessary to provide sufficient sensitivity. In our study, using a single antigen would have resulted in a drop of 3.3% in sensitivity, whilst the specificity would have increased by less than 1%. In another study that compared the diagnostic accuracy of the commercialized versions of both RDTs on stored serum samples from West Africa, the sensitivity of both tests was reported to be very good while the specificity was low [21]. This might be explained by geographic differences in the specificity of responses to VSG antigens but it may also suggest that results obtained using stored samples could differ from those obtained on fresh whole blood samples or alternatively, some changes in the buffer to optimize sensitivity could also modify specificity. In the present study, 16 cases would have been missed if only the prototype RDT had been used, and 9 if only CATT on whole blood had been used, reflecting the imperfect nature of both tests. Based on these observations, further optimization of the prototype RDT to improve both its sensitivity and specificity, which is critical when a test has to be used in situations of low prevalence, is recommended. The one case that was missed by CATT and RDT but found positive by CATT on diluted plasma could indicate the existence of a prozone effect, as has been reported with other serological tests [22].

Both of the antigens used in the prototype RDT are produced from pathogenic trypanosomes harvested from artificially infected rodents. Difficulties in their production could pose a challenge in scaling up production of the test. Altogether, both tests are imperfect, and reflect the difficulty of their use in settings of low prevalence and/or insufficient knowledge on parasite strain variation and/or the elicited immune reactions, highlighting the need for more investments in development of optimal tests. Recently, promising results were reported when prototype RDTs made using recombinant antigens were compared with the commercialized version of the SD BIOLINE HAT [23].

This study had a number of limitations. Firstly, the study lacked the level of statistical power that we had aimed for, and secondly, the results of the present study were not systematically validated with reference tests for antibody detection such as immune trypanolysis [24]. Thirdly, the mAECT test, which is the most sensitive parasitological method for *T*. *b*. *gambiense* HAT [25], was not routinely used in the CAR and was therefore not included in that part of the study, potentially leading to the misclassification of some infected individuals as controls. It is possible that participants who were reported as false positives with either serological test were in fact true positives, compromising in particular the reported specificity of the screening tests. Indeed both tests were least specific in the CAR. This is supported by the observation that when LAMP and PCR were performed on 70% of the seropositive but parasitologically negative samples, 3.14% were positive on both tests (S2 File). Most of the positive samples were from CAR where mAECT, was not performed. Approximately 8% of seropositive samples were positive by either LAMP or PCR, pointing to the possibility that these could have been true infections that were missed by the methods that are currently in clinical use. Indeed recent retrospective studies using samples from HAT patient have shown that both methods, which detect parasite DNA, have a sensitivity of up to 90% [25, 26, 28]. However, when human-infective parasite DNA is detected, it is unclear whether this should be taken as a marker of infection or disease; both spontaneous resolution and persistent latent infection can occur [21, 26]. Introduction of such molecular tests into clinical use may be a challenge and strategies on how they could be used to contribute to elimination of the disease have still to be fully explored [21, 26, 27, 28].

RDTs have a number of advantages, especially in settings of low prevalence and where the target is to eliminate the disease. They are simple, instrument-free, easy to use, and do not require a cold chain for storage. This study has demonstrated that performance of the SD BIOLINE HAT RDT is comparable to that of CATT 1/8. The recommendations arising from the study have since been used to optimize the prototype RDT, and performance and implementation studies using the optimized product are going on in several countries.

Introduction of HAT RDTs in routine use as an alternative to CATT could have significant cost implications on the healthcare system, which should be determined and the information used to guide policy decisions. While the benefits of using RDTs for passive screening in health facilities are evident, the cost-effectiveness of algorithms that use RDTs in active screening in place of CATT should be evaluated. During active screening using CATT for example, blood samples from 10 individuals are tested in parallel, which is advantageous because a large number of people can be tested in one day. When RDTs are used instead, one person is tested at a time, and the results are read after 15 minutes, meaning that fewer people are likely to be screened in one day, with a negative impact on population coverage. Similarly, the reagents used to perform the CATT test are packed in multiple doses while RDTs are single devices, which occupy a larger space during transportation, thus posing a challenge and additional costs to the mobile team. A detailed analysis of these aspects is currently being prepared as a follow on publication.

## Supporting Information

S1 File Comparison of the performance of antigens in a prototype HAT rapid diagnostic test (RDT) with CATT using stored samples. (DOCX)

S2 File Proportion of positive and negative test results using molecular analysis (LAMP and PCR). (DOCX)

S3 File STARD checklist. (DOC)
