# Supplementary material for: Sensitivity and Specificity of a Prototype Rapid Diagnostic Test for the Detection of Trypanosoma brucei gambiense Infection: A Multi-centric Prospective Study
Source: PLoS Negl Trop Dis. 2016 Apr 8;10(4):e0004608. doi: 10.1371/journal.pntd.0004608 (PMC4825971; doi:10.1371/journal.pntd.0004608)
Supplement: S2 File — (DOCX) [file pntd.0004608.s002.docx]

|  |  |  |  |  |
| --- | --- | --- | --- | --- |
|  | LAMP - & PCR - | LAMP + & PCR - | LAMP - & PCR + | LAMP + & PCR + |
| RDT + & CATT - | 412 | 33 | 44 | 6 |
| RDT - & CATT + | 41 | 3 | 5 | 4 |
| RDT + & CATT + | 211 | 29 | 23 | 16 |
|  |  |  |  |  |
| **Total** | **664** | **65** | **72** | **26** |
| **%** | **80.29%** | **7.86%** | **8.71%** | **3.14%** |
|  |  |  |  |  |

S2 file: Proportion of positive and negative test results using molecular analysis (LAMP and PCR). A subgroup of 70 % of the samples from the cohort that were negative by parasitology but positive with at least one serological test.

RDT: Rapid diagnostic test for HAT

CATT: Card agglutination test for trypanosomiasis

LAMP: Loop mediated isothermal amplification

PCR: Polymerase chain reaction
