# Supplementary material for: Sensitivity and Specificity of a Prototype Rapid Diagnostic Test for the Detection of Trypanosoma brucei gambiense Infection: A Multi-centric Prospective Study
Source: PLoS Negl Trop Dis. 2016 Apr 8;10(4):e0004608. doi: 10.1371/journal.pntd.0004608 (PMC4825971; doi:10.1371/journal.pntd.0004608)
Supplement: Supplementary file 3 [file pntd.0004608.s001.docx]

Comparison of the performance of antigens in a prototype HAT RDT with CATT using stored samples. VSG LiTat 1.3 corresponds to band 1 and VSG LiTat 1.5 to band 2 on the prototype RDT.

Study site: International Livestock Research Institute, Nairobi, Kenya

Samples: Plasma from 150 HAT patients and 150 controls (WHO Specimen Bank)
